# Chloroplast whole genome assembly and phylogenetic analysis of *Persicaria criopolitana* reveals its new taxonomic status

**DOI:** 10.1038/s41598-025-02686-5

**Published:** 2025-06-06

**Authors:** Tao Zou, Dan Li, Chun-Yan Zhao, Ming-Lin Chen

**Affiliations:** College of Life Sciences, Anhui Normal University, Wuhu, 241000 PRC China

**Keywords:** Plant molecular biology, Genome

## Abstract

*Persicaria criopolitana* (Polygonaceae), a dominant annual herb in wetland ecosystems, is ecologically and horticulturally significant. Despite its prevalence, genomic resources for clarifying its phylogenetic relationships and supporting conservation efforts remain limited. The complete chloroplast genome of *P. criopolitana* was sequenced, assembled, and annotated. Comparative genomic analyses with other *Persicaria* species were conducted to identify structural variations and evolutionary dynamics. Phylogenetic relationships were reconstructed using maximum-likelihood methods based on whole chloroplast genome sequences. The chloroplast genome (159,427 bp) exhibits a conserved quadripartite structure, encoding 131 genes, including 86 protein-coding, 37 tRNA, and 8 rRNA genes. Key features include: 208 simple sequence repeats (SSRs) were detected, predominantly mononucleotide motifs. A pronounced preference for A/U-ending codons, with leucine as the most frequent amino acid. A 62-bp extension of the *ndhF* gene into the inverted repeat (IRb) region within the small single-copy (SSC) region.Phylogenetic resolution: *P. criopolitana* clusters within *Persicaria* sect. *Polygonum*, demonstrating distant divergence from sect. *Cephalophilon*.This study provides the first complete chloroplast genome resource for *P. criopolitana*, resolving its taxonomic position and revealing adaptive genomic signatures. These findings advance molecular tools for species identification, inform conservation strategies, and elucidate evolutionary mechanisms in *Persicaria*.

## Introduction

*Persicaria*, a genus within the Polygonaceae family, has been reclassified from its original grouping, now encompassing approximately 150 species^1^, predominantly found in the northern temperate zone, with some species in Africa, India, and subtropical regions of South America^2^. The genus is categorized into four groups: Sect. *Cephalophilon*, Sect. *Echinocaulon*, Sect. *Polygonum* and Sect.*Tovara*^1^. Notably, three species initially classified under Sect.*Tovara* were later reassigned to *Persicaria*. *Persicaria criopolitana*, an annual herb, is classified under Sect. *Cephalophilon* due to its capitular inflorescence^3^. It is stoloniferous, reaching 10–15 cm in height, with lanceolate leaves measuring 1–3 cm. The plant features a terminal capitulum with a reddish, deeply divided perianth, five stamens with purplish-red anthers, and a bifurcated style. *P. criopolitana* typically inhabits freshwater zones where terrestrial and aquatic environments converge. Current research on *P. criopolitana* primarily addresses its reproductive traits^4^, ecological significance^5^, and morphology^6–9^. The study demonstrates distyly in *P. criopolitana*, while the morphological diversity of sect. *Cephalophilon* (encompassing achenes, leaves, floral structures, and pollen morphology) provides multidimensional evidence supporting its monophyly, revealing significant morphological differentiation from sect. *Polygonum*. In investigations of floral morphology within sect. *Cephalophilon*, researchers have proposed reclassification adjustments for certain species based on taxonomic evaluations.However, studies on the chloroplast genome’s structure, genetic characteristics, and sequence analysis remain unpublished.

Chloroplasts, a type of plastid, are prevalent in land plants, algae, and certain protists, serving as essential organelles with independent genetic material^10^. Photosynthesis occurs within chloroplasts, providing a crucial energy source for the evolution of early life^11–13^. Comprehensive knowledge of the chloroplast genome and its evolutionary role is vital for advancing the exploration and utilization of chloroplast functions^14,15^.

Chloroplast genomes are predominantly circular, with few exceptions being linear. Their sizes vary significantly: microtubule plants typically range from 120 to 160 Kb^16^, ferns around 160 Kb^17–19^, and algae from 37 Kb to 2 Mb^20^. Generally stable, chloroplast genomes exhibit a quadripartite structure comprising a Large Single Copy (LSC) region, a Small Single Copy (SSC) region, and two Inverted Repeat regions (IRA and IRB)^21,22^. However, some plants, such as Fabaceae^23^, Cactaceae^24,25^, and certain algae^26^, lack these large inverted repeats.

In this study, the chloroplast genome was sequenced, assembled and annotated, and its codon preference, repeat sequence, IR boundary and phylogeny were analyzed.

## Results

### Chloroplast genome structure

**Fig. 1:**
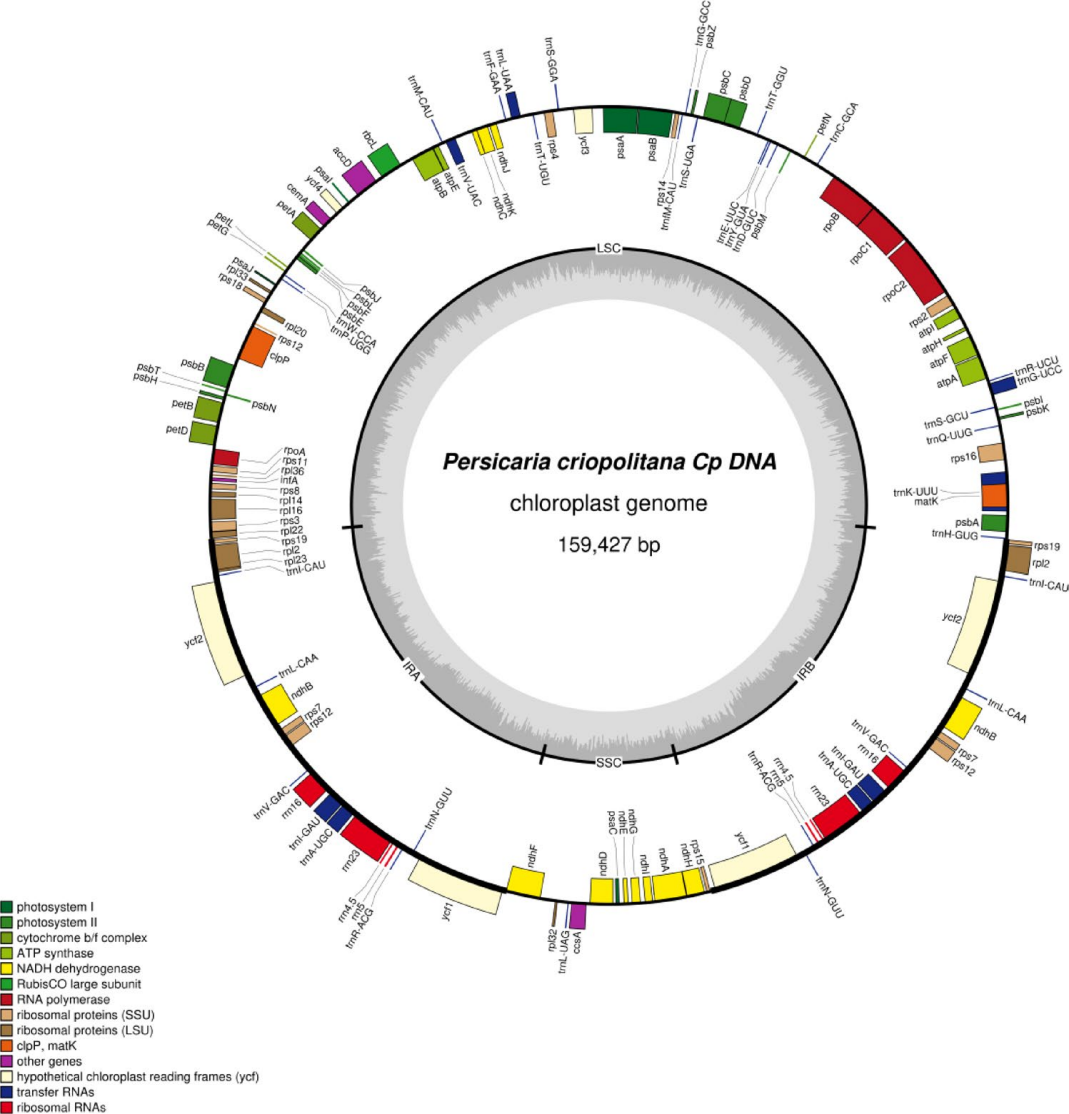
Chloroplast genome map of *P. criopolitana*. Genes shown inside the circle are transcribed clockwise, whereas genes outside are transcribed counterclockwise. Te light gray inner circle shows the AT content, the darkgray corresponds to the GC content.

The *P. criopolitana* chloroplast genome spans 159,427 bp, with a total base count of 5,627,492,462, exhibiting a typical tetrad structure (Fig. 1). This structure comprises four regions: LSC (83,995 bp), SSC (13,140 bp), IRA (31,146 bp), and IRB (31,146 bp). The genome’s GC content is 38.25%, while AT content is 61.75%, indicating a preference for A and T bases. Notably, the GC content in the IR regions (41.46%) exceeds that of the LSC (36.64%) and SSC (33.32%) regions.The chloroplast genome of *P. criopolitana* comprises 131 annotated genes, including 86 protein-coding genes (Coding sequence: CDS), 37 tRNA genes, and 8 rRNA genes. These genes are categorized into four functional groups: photosynthesis-related genes, self-replication genes, other genes, and genes of unknown function. Introns must be excised during transcription, influencing gene expression rates. Analysis of chloroplast genes indicates that *ndhA*, *ndhB*, *petB*, *petD*, *atpF*, *rpl16*, *rpl2*, *rps16*, *rpoC1*, *trnA-UGC*, *trnG-UCC*, *trnI-GAU*, *trnK-UUU*, *trnL-UAA*, *trnV-UAC*, and *ycf3* each contain one intron, whereas *rps12* and *clpP* contain two introns each. The *P. criopolitana* chloroplast genome comprises 18 double-copy genes, representing 18.74% of the total. These include one NADH dehydrogenase subunit gene (*ndhB*), one large ribosomal subunit protein gene (*rpl2*), three small ribosomal protein genes (*rps12*, *rps19*, *rps7*), four ribosomal RNA genes (*rrn16*, *rrn23*, *rrn4.5*, *rrn5*), seven transfer RNA genes (*trnA-UGC*, *trnI-CAU*, *trnI-GAU*, *trnL-CAA*, *trnN-GUU*, *trnR-ACG*, *trnV-GAC*), and two conserved open reading frames (*ycf1h*, *ycf2*). Additionally, the genome includes four genes of unknown function (*ycf1*, *ycf2*, *ycf3*, *ycf4*) and lacks pseudogenes (Table 1).

### Repeat sequence analysis

**Fig. 2:**
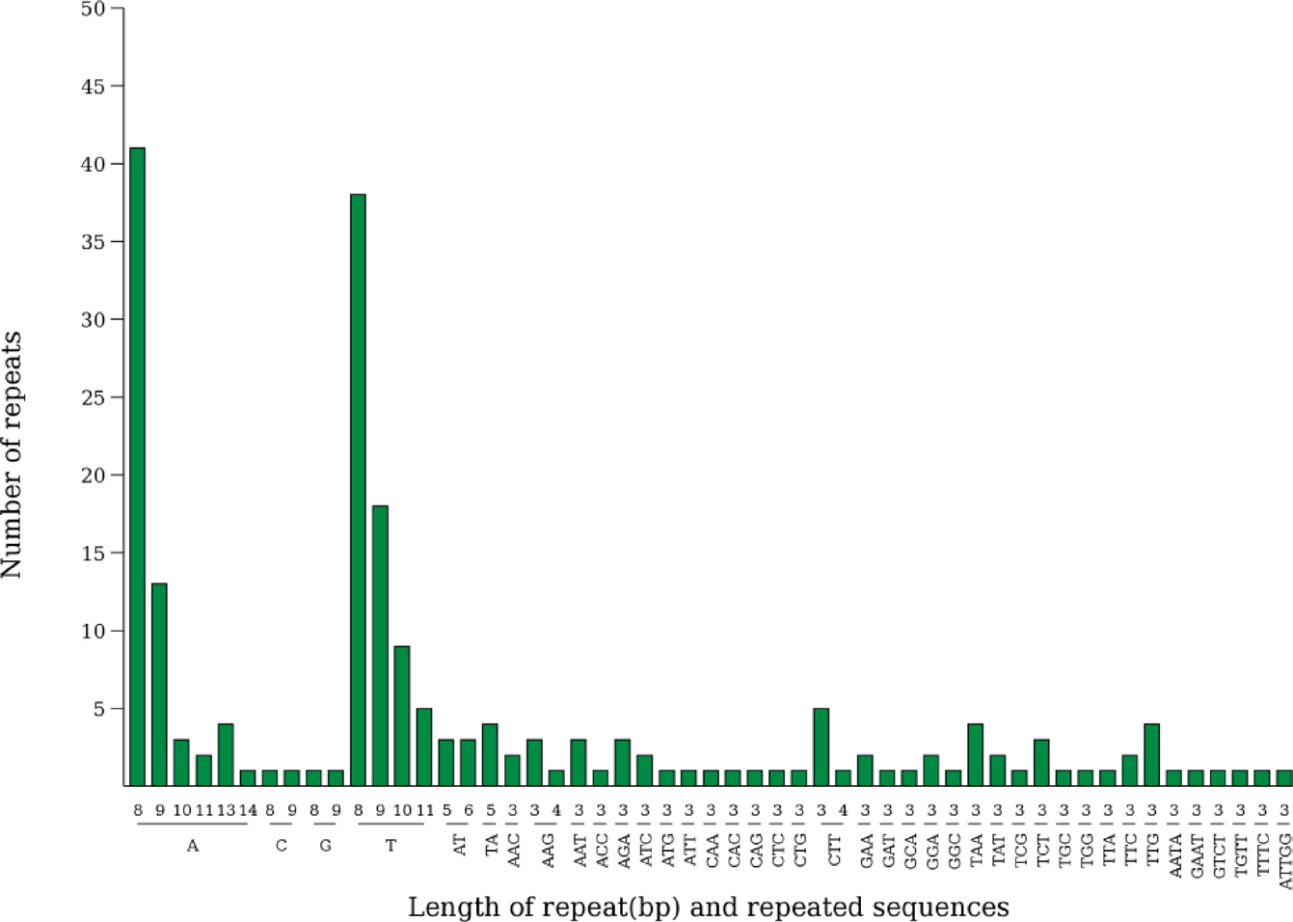
SSR type and number of P. criopolitana chloroplast genome, The x-axis represents SSR repeat units, and the y-axis represents the number of repeat units.

**Fig. 3:**
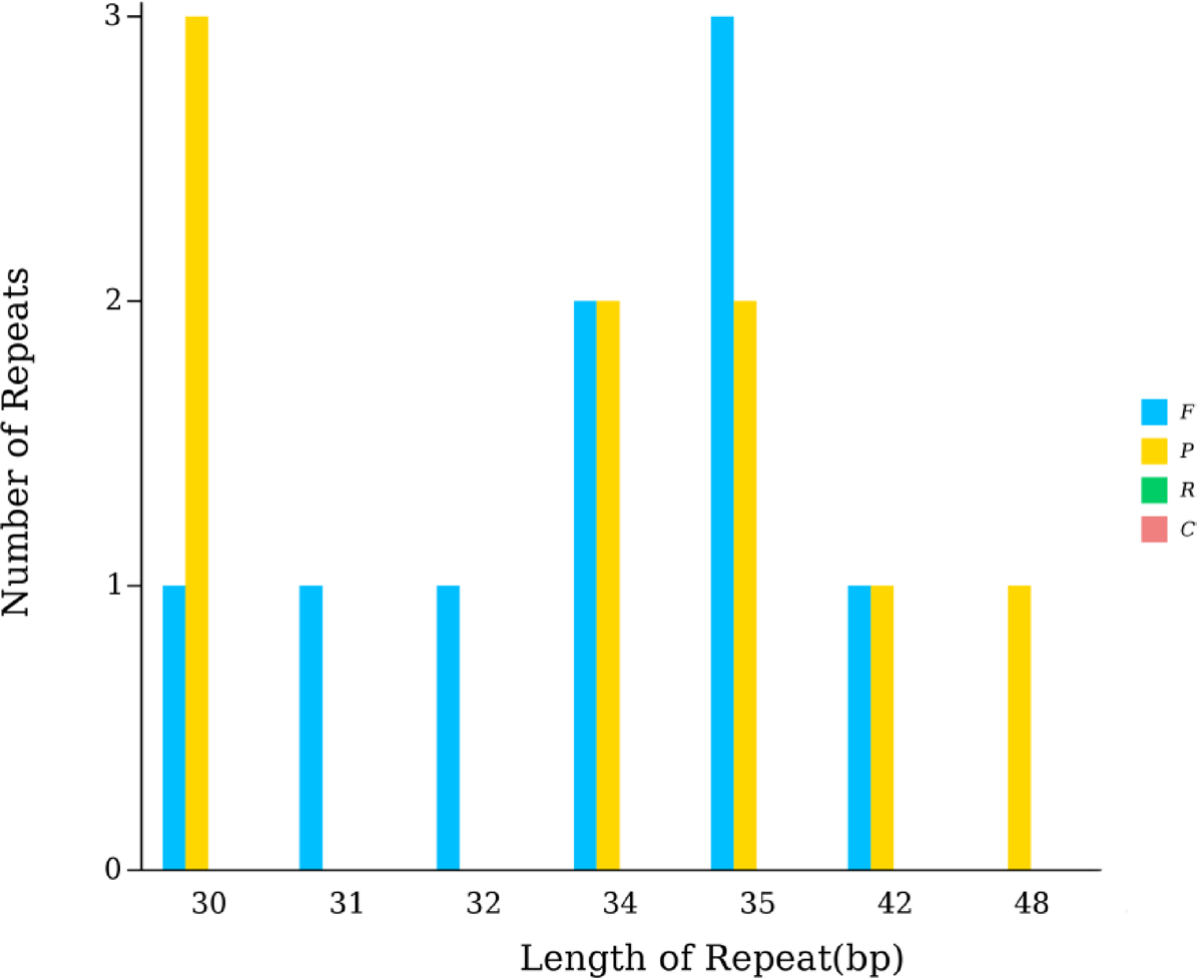
Tandem repeats type and number of *P. criopolitana* chloroplast genome, The x-axis represents the length of scattered repetitive sequences, and the y-axis represents the quantity of scattered repetitive sequences. F stands for forward repeats, P for palindromic repeats, R for reverse repeats, and C for complementary repeats.

The chloroplast genome of *P. criopolitana* contains 208 Simple Sequence Repeats (SSRs) loci, distributed across 112 loci in the LSC region, 26 in the SSC region, and 70 in the IR region. These SSRs include 138 mononucleotide, 10 dinucleotide, 54 trinucleotide, 5 tetranucleotide, and 1 pentanucleotide repeats (Fig. 2). The most frequently repeated bases were A/T, followed by AT/TA. Notably, 156 SSRs, or 75% of the total, were composed of A and T bases, indicating a dominance and preference for these bases. Eighteen tandem repeats were identified, comprising 9 forward repeats and 9 palindromic repeats, with lengths spanning 30 to 48 bp(Fig. 3.), predominantly situated in the LSC and IR regions. No complementary or reverse repeats were found in the chloroplast genome of *P. criopolitana*.

### Codon bias analysis

**Fig. 4:**
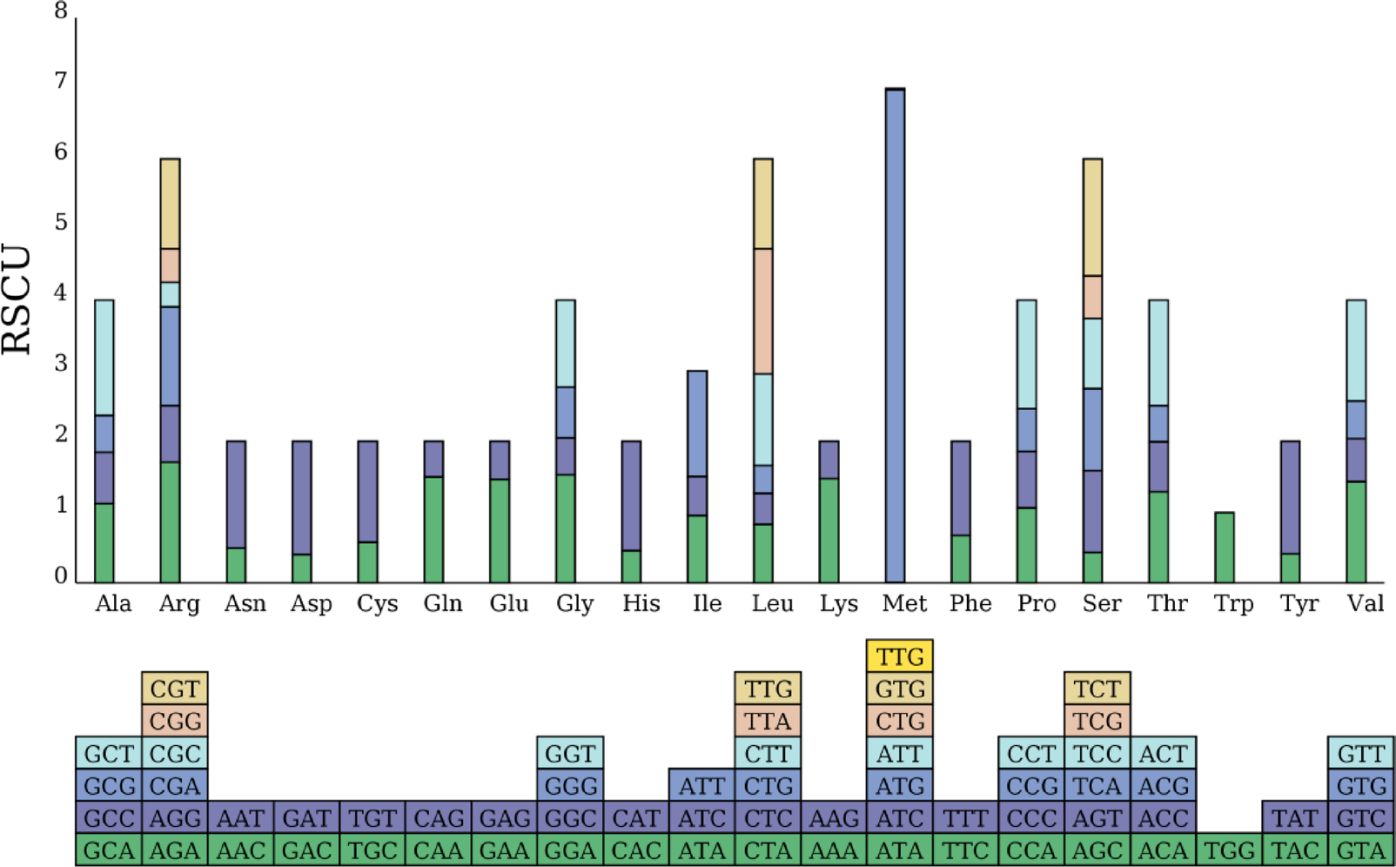
Relative synonymous codon usage (RSCU) for protein-coding genes in *P. criopolitana*.The blocks below represent all codons encoding each amino acid, and the height of the bars above represents the sum of the RSCU values for all codons.

The chloroplast genome of *Persicaria criopolitana* contains 28,116 codons across 66 types (Fig. 4.). The termination codons identified are UAA, UAG, and UGA, with UAA being the most prevalent. Codons for leucine (Leu) are the most frequent, numbering 2,960 and constituting approximately 10.53% of the total. In contrast, cysteine (Cys) is encoded by only 302 codons, representing about 1.07%. The 66 codons encode 20 amino acids. Codon usage analysis indicates that six codons exhibit strong preference (RSCU ≥ 1.60): UAA, GCU, AGA, UCU, UUA, and AUG. Meanwhile, 34 codons show weak preference (RSCU < 1.00), and UGG displays no bias (RSCU = 1). Of the 31 high-frequency codons (RSCU > 1.00), 29 end in A or U, comprising roughly 93.55% of these codons. Only two high-frequency codons, AUG and UUG, end in G, and none end in C.

**Table 1:** Gene composition of *P. criopolitana* Chloroplast genome. gene*: gene with one introns; gene**: gene with two introns; #Gene: Pseudo gene; gene(2): Number of copies of multi-copy genes.

Category	Gene group	Gene name
Photosynthesis	Subunits of photosystem I	*psaA*,* psaB*,* psaC*,* psaI*,* psaJ*
	Subunits of photosystem II	*psbA*,* psbB*,* psbC*,* psbD*,* psbE*,* psbF*,* psbH*,* psbI*,* psbJ*,* psbK*,* psbL*,* psbM*,* psbN*,* psbT*,* psbZ*
	Subunits of NADH dehydrogenase	ndhA*, ndhB*(2), *ndhC*,* ndhD*,* ndhE*,* ndhF*,* ndhG*,* ndhH*,* ndhI*,* ndhJ*,* ndhK*
	Subunits of cytochrome b/f complex	*petA*, petB*, petD*,* petG*,* petL*,* petN*
	Subunits of ATP synthase	*atpA*,* atpB*,* atpE*, atpF*,* atpH*,* atpI*
	Large subunit of rubisco	*rbcL*
	Subunits photochlorophyllide reductase	*-*
Self-replication	Proteins of large ribosomal subunit	*rpl14*, rpl16*, rpl2*(2), *rpl20*,* rpl22*,* rpl23*,* rpl32*,* rpl33*,* rpl36*
	Proteins of small ribosomal subunit	*rps11*, rps12**(2), *rps14*,* rps15*, rps16*,* rps18*,* rps19*(2), *rps2*,* rps3*,* rps4*,* rps7*(2), *rps8*
	Subunits of RNA polymerase	*rpoA*,* rpoB*, rpoC1*,* rpoC2*
	Ribosomal RNAs	*rrn16*(2), *rrn23*(2), *rrn4.5*(2), *rrn5*(2)
	Transfer RNAs	trnA-UGC*(2), *trnC-GCA*,* trnD-GUC*,* trnE-UUC*,* trnF-GAA*,* trnG-GCC*, trnG-UCC*,* trnH-GUG*,* trnI-CAU*(2), trnI-GAU*(2), trnK-UUU*,* trnL-CAA*(2), trnL-UAA*,* trnL-UAG*,* trnM-CAU*,* trnN-GUU*(2), *trnP-UGG*,* trnQ-UUG*,* trnR-ACG*(2), *trnR-UCU*,* trnS-GCU*,* trnS-GGA*,* trnS-UGA*,* trnT-GGU*,* trnT-UGU*,* trnV-GAC*(2), trnV-UAC*,* trnW-CCA*,* trnY-GUA*,* trnfM-CAU*
Other genes	Maturase	*matK*
	Protease	clpP**
	Envelope membrane protein	*cemA*
	Acetyl-CoA carboxylase	*accD*
	c-type cytochrome synthesis gene	*ccsA*
	Translation initiation factor	*infA*
	other	*-*
Genes of unknown function	Conserved hypothetical chloroplast ORF	*ycf1*(2), *ycf2*(2), ycf3*,* ycf4*

### Comparative analysis of IR boundaries

**Fig. 5:**
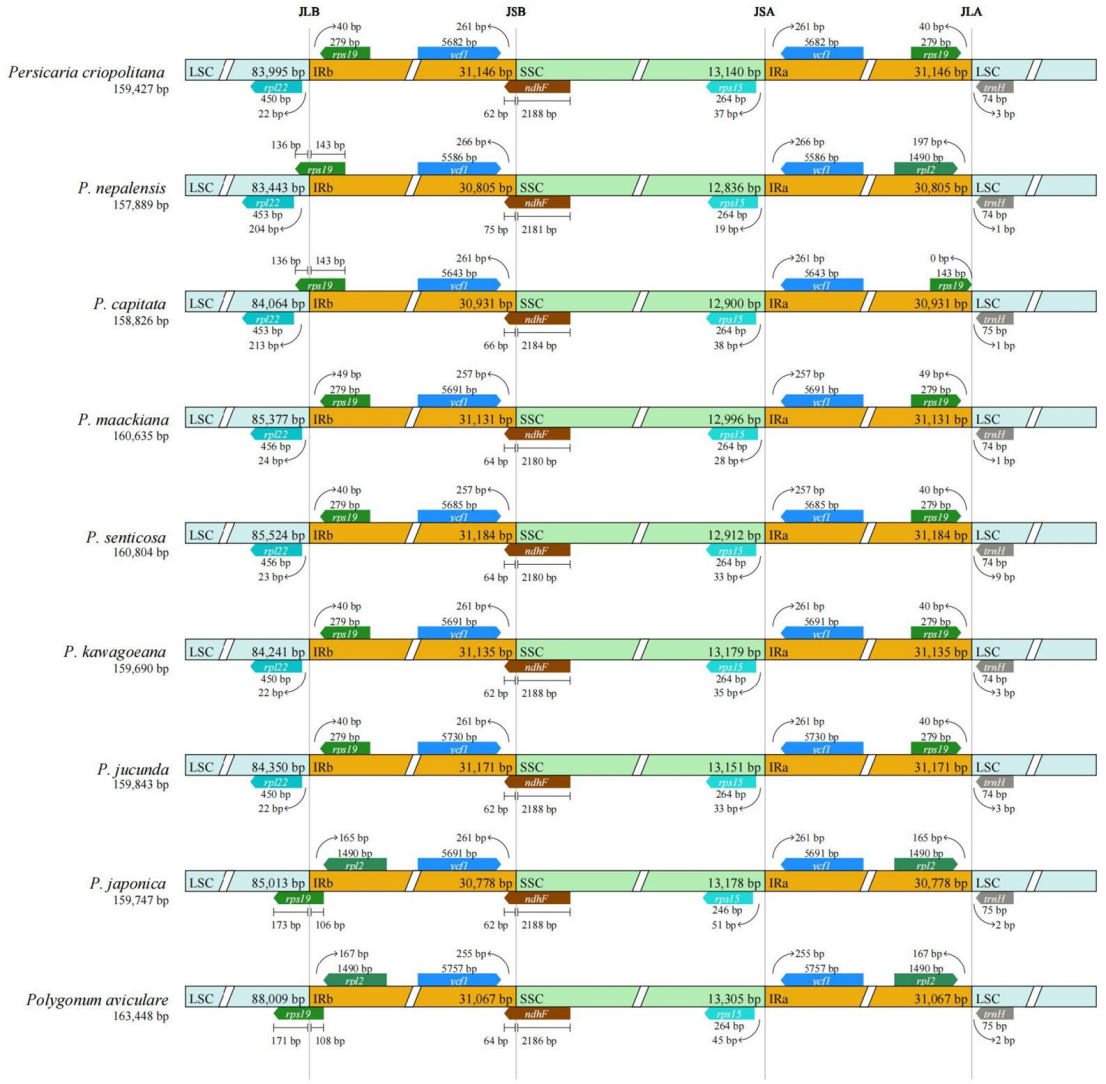
Changes of IR/SC boundary of chloroplast genomes of nine Polygonaceae species.

The chloroplast genomes of nine Polygonaceae species, including *P. criopolitana*, were analyzed, revealing genome lengths ranging from 157,889 bp in *P. nepalensis* to 163,448 bp in *Polygonum aviculare*(Fig. 5). The inverted repeat (IR) regions in all species were approximately 31 Kb. The boundary genes at the JLB and JLA regions were generally consistent across species. For JLB, the flanking genes were typically *rpl22*, *rps19*, and *rpl2*, except in *P. japonica* and *Polygonum aviculare*, which had *rps19* and *rpl2*. In *P. criopolitana* and six other species, the genes were *rpl22* and *rps19*. For JLA, the flanking genes included *rps19*, *rpl2*, and *trnH*, with *rpl22* and *rps19* in *P. nepalensis*, *P. japonica*, and *Polygonum aviculare*. In contrast, *P. criopolitana* and five other species had *rps19* and *trnH*, except for *P. nepalensis*, *P. japonica*, and *Polygonum aviculare*, where the genes were *rpl2* and *trnH*. The nine species exhibited no variation in the flanking gene types of JSB and JSA, specifically *vcf1* and *ndhF*; *rps15* and *vcf1*, respectively. In *P. criopolitana*, the *ndhF* gene measured 2250 bp, with 62 bp extending into the IRb region. This study’s findings for *P. criopolitana* aligned with those for *P. nepalensis* and *P. capitata* in terms of boundary gene types, although there were differences in gene boundary distances. Notably, the *ndhF* gene in the SSC region expanded by 62 bp into the IRb. The flanking genes in JLB were *rpl22* and *rps19*, while in JLA, they were *rps19* and *trnH*. This differs from some species and is likely due to the IR’s expansion into the LSC.

### Phylogenetic analysis

**Fig. 6:**
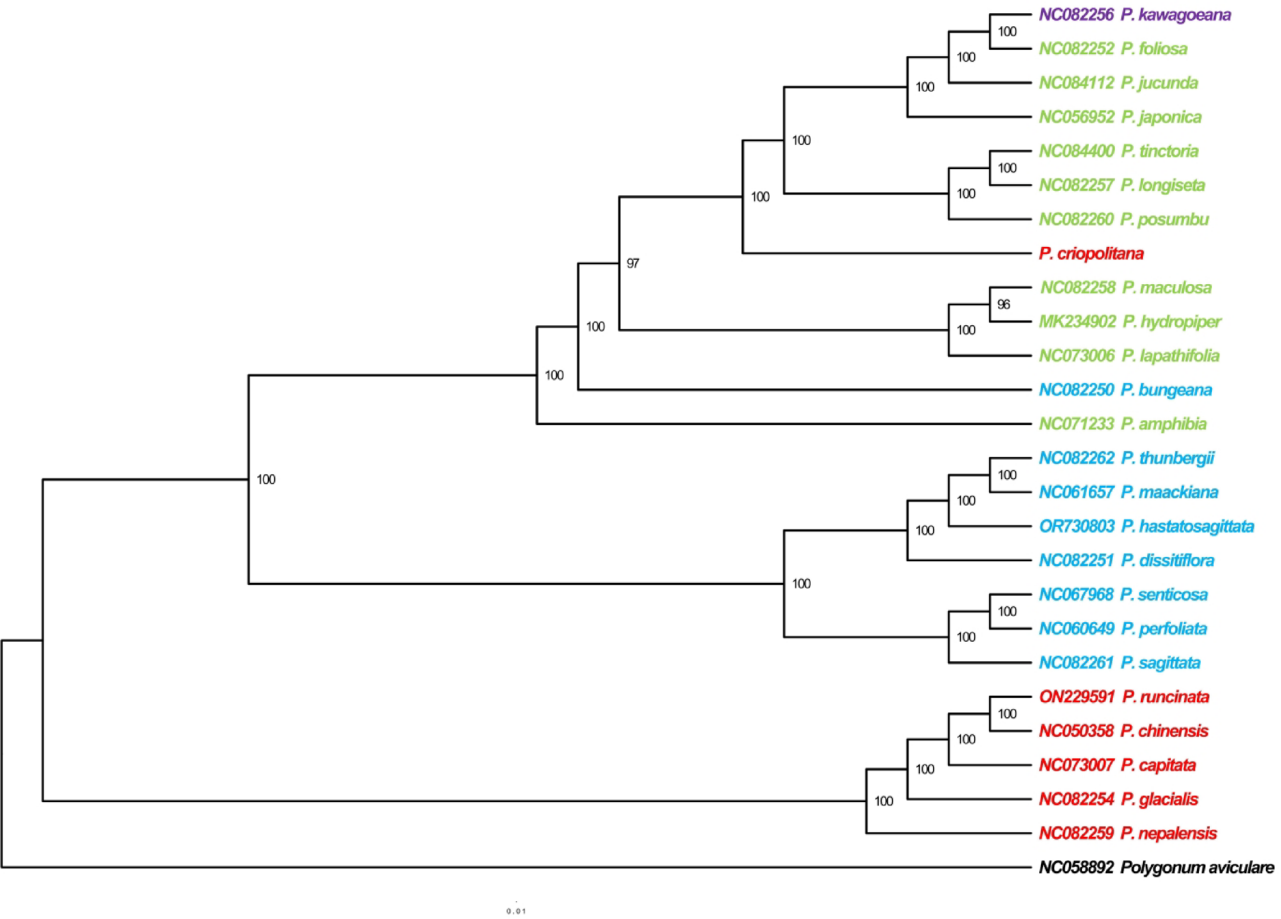
Phylogenetic analysis utilizing chloroplast genome sequences identifies *Polygonum aviculare* as the outgroup (black). Sect. *Cephalophilon* is denoted in red, Sect. *Echinocaulon* in blue, Sect. *Polygonum* in green, and *Persicaria kawagoeana* in purple.

The evolutionary relationships of *Persicaria* species were examined using *polygonum aviculare* as an outgroup (Fig. 6). Phylogenetic trees, constructed via the maximum likelihood method from chloroplast whole-genome data of 26 species, exhibited strong support, with branch node support exceeding 95%. The phylogenetic analysis revealed that the Sect. *Cephalophilon* of the *Persicaria* genus diverged first, followed by the Sect. *Echinocaulon* and Sect. *Polygonum*. The 25 *Persicaria* species were categorized into three clades, with one clade comprising *P. kawagoeana*, *P. criopolitana*, *P. bungeana*, and the Sect. *Polygonum*. Sect. *Echinocaulon* and Sect. *Cephalophilon* represent distinct branches, contrary to the *Flora of China* records. *P. kawagoeana*, *P. foliosa*, *P. jucunda*, *P. japonica*, *P. tinctoria*, *P. longiseta*, and *P. posumbu* form a clade sister to *P. criopolitana*, with full nodal support. The phylogenetic analysis indicates that *P. criopolitana* is distantly related to Sect. *Cephalophilon* but closely related to Sect. *Polygonum*.

## Discussion

In this study, the chloroplast genome of *P. criopolitana* was sequenced and analyzed using second-generation techniques. The genome exhibited a typical quadripartite structure, measuring 159,427 bp with a GC content of 38.25%. It contained 131 annotated genes, comprising 86 protein-coding genes, 37 tRNAs, and 8 rRNAs. These findings align with the known characteristics of the Polygonum chloroplast genome.

Repeat sequences in a genome are identical or similar DNA segments occurring at various genomic locations. These sequences play diverse roles, including gene regulation, chromosome structure maintenance, and evolutionary variation^27–30^. Based on distribution, they are categorized into simple and dispersed repeat sequences. In the chloroplast genome of *P. criopolitana*, 208 SSR sites were identified, distributed across the LSC, SSC, and IR regions, with a predominance of single nucleotide repeats (138) and no repeats of six or more nucleotides. Notably, 156 SSRs were composed of A and T bases, indicating a base preference and accounting for 75% of the repeats. Additionally, 18 tandem repeats were identified, comprising 9 forward and 9 palindromic repeats, primarily located in the LSC and IR regions.

Due to codon degeneracy, each amino acid is encoded by at least one and up to eight codons. Codon usage varies significantly across different species and organisms, a phenomenon known as codon bias. This bias results from a combination of natural selection, mutation, and genetic drift^31–33^. The Relative Synonymous Codon Usage (RSCU) quantifies codon preference by comparing the observed frequency of a codon to its expected frequency. An RSCU of 1 indicates no preference, while RSCU values greater than 1 suggest strong preference, and values less than 1 indicate weak preference. In the chloroplast genome of *P. criopolitana*, six codons (UAA, GCU, AGA, UCU, UUA, and AUG) exhibit strong preference with RSCU values of 1.60 or higher. Additionally, 31 codons have high frequency usage (RSCU > 1), with 29 ending in A or U, suggesting a preference for A and U at the third codon position, consistent with chloroplast genomes of other higher plants^34^.

Analyzing the IR boundaries of chloroplast genomes is crucial for understanding their structure and evolutionary dynamics^35^. The IR region is typically a conserved sequence within the chloroplast genome, and its contraction or expansion often leads to gene rearrangements^36^, thereby influencing genomic stability. Comparative analysis of IR boundary features across different plant species can elucidate phylogenetic relationships. In *P. criopolitana*, the IR region measures approximately 31 Kb, similar to most *Persicaria* species, with notable differences in boundary genes, particularly at JLB and JLA. The JLB flanking genes are *rpl22* and *rps19*, while those for JLA are *rps19* and *trnH*. The JSB flanking genes are *vcf1* and *ndhF*, and for JSA, they are *rps15* and *vcf1*. Notably, *ndhF*, located in the SSC, extends into IRb by 62 bp. Based on the IR boundary, *P. criopolitana* shows significant differentiation from other species in Sect. *Cephalophilon*, while it is less differentiated from species in Sect. *Echinocaulon* and Sect. *Polygonum*.

Chloroplast genomes are crucial for phylogenetic analysis in plants due to their conserved nature and minimal genome rearrangements, making them effective for studying plant phylogenetic relationships^37,38^. To elucidate the phylogenetic position and relationships of *P. criopolitana* within the *Persicaria* genus, a study of 25 *Persicaria* species revealed that *P. criopolitana* and the Sect. *Polygonum* group clustered into a single branch, distinct from the Sect. *Cephalophilon*. In *Flora of China*, *P. criopolitana* is classified under Sect. *Cephalophilon* due to its capitular inflorescence. This finding diverges from the classification in *Flora of China* but aligns with results from IR boundary analysis. The comparative analysis of the chloroplast genome and phylogenomic investigations within *P. criopolitana* and its congeneric taxa have not only elucidated the evolutionary dynamics and molecular mechanisms governing the diversification and ecological adaptation of Polygonaceae species, but also established a robust genetic framework for developing targeted conservation strategies. To advance the practical implementation of these findings, future research should integrate multidimensional datasets encompassing genomics, transcriptomics, and metabolomics with advanced ecological niche modeling approaches, thereby enhancing the effective utilization of this taxon’s biological characteristics in conservation biology and ecosystem management.

## The collapse of neutral theory and implications for evolutionary studies

The conclusions of this study must be interpreted with caution within the context of an ongoing paradigm shift in molecular evolutionary theory. The neutral theory of molecular evolution, long regarded as foundational in evolutionary genetics, has been systematically invalidated by empirical evidence. Despite this, its methodological legacy persists, necessitating a critical reassessment of current analytical frameworks.

The neutral theory’s core premise-that molecular evolution is predominantly driven by random genetic drift-relied heavily on the molecular clock hypothesis. This hypothesis erroneously attributed the genetic equidistance phenomenon to time-dependent accumulation of neutral mutations. However, proteomic analyses demonstrate that equidistance patterns reflect functional constraints rather than neutral drift^39^. The collapse of the molecular clock hypothesis directly undermines the theoretical foundation of neutrality, yet paradoxically, phylogenetic methodologies remain anchored to its assumptions.

Accumulating evidence decisively refutes key predictions of the neutral theory: Over 90% of human mitochondrial genome sites exhibit strong purifying selection, with synonymous mutations demonstrating significant disease associations^40^. These findings contradict the neutralist assumption of nonfunctional “junk DNA. “Short tandem repeats (STRs), previously considered evolutionarily neutral, modulate transcriptional activity by directly binding transcription factors (TFs), with length variations altering gene expression by up to 70-fold. Disease-associated STR variants highlight their functional significance^41^. Lynch et al. (2024) revealed that mutation loads in natural populations exceed neutral theory predictions by orders of magnitude, with purifying selection operating across 85% of eukaryotic genomes^42^.

The emerging Maximum Genetic Diversity Theory (MGD) provides a robust alternative framework, proposing that genetic diversity is constrained by an upper limit imposed through natural selection. This limit is mediated by two key mechanisms: Preconfigured genomic architectures (e.g., gene regulatory networks) restrict mutation accumulation to maintain adaptive potential. DNA methylation and noncoding RNAs mitigate fitness costs of genetic variation, enabling diversity accumulation within selective boundaries^39,43^.

Current phylogenetic methodologies remain compromised by residual neutral assumptions: Metrics such as dN/dS ratios erroneously treat synonymous mutations as neutral, despite their demonstrated phenotypic associations^40^. Divergence time estimates persist in employing discredited molecular clock frameworks.

In conclusion, while methodologically entrenched, the neutral theory is fundamentally irreconcilable with empirical reality. As with most contemporary studies in this field, our findings remain provisional until methodologies align with post-neutral frameworks. A paradigm centered on functional constraints and epigenetic complexity—rather than neutral drift—is imperative to unraveling the logic of evolutionary processes.

## Materials and methods

### Sample collection

In this study, we collected young leaves from Wuhu City, Anhui Province, China (coordinates: N 118°20′5″, E 31°17′28″; altitude: 13 m), identified as *Persicaria criopolitana* by Prof. Chen Minglin of Anhui Normal University. The samples were rapidly frozen in liquid nitrogen and stored in an ultra-low temperature freezer for chloroplast genome sequencing and analysis. Voucher specimens (specimen no: 2022ASD96062407) are preserved in the Herbarium of the College of Life Sciences, Anhui Normal University. Collection adhered to the Regulations of the People’s Republic of China on Wild Plant Protection, with authorization from local forestry authorities and the Grassland Bureau of Anhui Province, China.

### Extraction and sequencing of Chloroplast DNA

Following genomic DNA testing, the DNA molecules are fragmented using ultrasound technology. These fragments undergo purification, end modification, addition of 3’-end A bases, and are ligated to sequencing adapters. Size selection via agarose gel electrophoresis is performed, followed by PCR amplification to create a sequencing library. The libraries are quality-tested, and only those meeting standards are sequenced using the Illumina NovaSeq 6000^44^ platform with a 150 bp paired-end read length, yielding approximately 5 GB of raw data. The fastp v0.23.4^45^ software (https://github.com/OpenGene/fastp) is employed to filter these data, resulting in 18,634,081 high-quality reads with Q20 and Q30 ratios of 98.57% and 95.82%, respectively. These reads facilitate the subsequent assembly and annotation of the Polygonum chinensis chloroplast genome.

### Chloroplast genome assembly

Core modules employ SPAdes v3.10.1^46^ (http://cab.spbu.ru/software/spades/) for assembling the chloroplast genome, utilizing k-mers of 55, 87, and 121, independent of a reference genome. Step 1 involves using SPAdes to assemble the cpDNA sequence, yielding the SEED sequence of the chloroplast genome. In Step 2, k-mer iteration extends the seed. If this results in a contig, it is designated as the pseudo-genome sequence, proceeding directly to Step 6. Step 3 utilizes SSPACE v2.0^47^ (https://github.com/nsoranzo/sspacebasic) to connect contig sequences from Step 2 into scaffolds. Step 4 employs Gapfiller v2.1.1^48^ (https://sourceforge.net/projects/gapfiller/) to fill gaps in the scaffolds obtained in Step 3. Step 5 involves designing primers, performing PCR sequencing, and reassembling if gaps persist, until a complete pseudo-genome sequence is achieved. Step 6 compares the sequencing sequence to the pseudo-genome for correction. Finally, Step 7 corrects the pseudo-genome and rearranges coordinates based on chloroplast structure to produce the complete chloroplast circular genome sequence.

### Chloroplast gene structure annotation

Two methods were employed to enhance the accuracy of chloroplast genome annotation. Initially, Prodigal v2.6.3^49^ (https://www.github.com/hyattpd/Prodigal) was utilized to annotate chloroplast CDS, while HMMER v3.1b2^50^ (http://www.hmmer.org/) was used for rRNA prediction, and ARAGORN v1.2.38^51^ (http://www.ansikte.se/ARAGORN/) for tRNA prediction. Subsequently, gene sequences from related species available on NCBI were extracted and aligned against the assembly sequence using BLAST v2.6^52^ (https://blast.ncbi.nlm.nih.gov/Blast.cgi) to obtain a second set of annotation results. These two sets of annotations were then manually reviewed to resolve discrepancies, eliminate errors and redundancies, and define multi-exon boundaries, resulting in the final annotation. The chloroplast genome was visualized using OGDRAW^53^ (https://chlorobox.mpimp-golm.mpg.de/OGDraw.html).

### Chloroplast genome characterization and boundary comparison

MISA v1.0^54^ (MIcroSAtellite identification tool, https://webblast.ipk-gatersleben.de/misa/) was employed to analyze cpSSR using the parameters: 1–8 (≥ 8 repetitions of a single base), 2–5, 3–3, 4 − 3, 5 − 3, and 6 − 3. Duplicate sequences were identified using Vmatch v2.3.0^55^ (http://www.vmatch.de/) with a Perl script, setting parameters to a minimum length of 30 bp and a Hamming distance of 3, across four identification forms: forward, palindromic, reverse, and complement. A custom Perl script was used to filter unique CDS (selecting one from multiple copies) and perform codon preference analysis. The newly sequenced Polygamum L. leaves were compared to eight Polygamaceae species from the NCBI database (accession numbers: NC082259, NC073007, NC061657, NC067968, NC082256, NC084112, NC056952, NC058892). The chloroplast genome sequence was analyzed using the Genepioneer platform (http://112.86.217.82:9929) for boundary differences, and a comparative analysis chart was generated.

### Phylogenetic analysis

According to the *Flora of China*, *Persicaria* is classified into Sect. *Polygonum*, Sect. *Echinocaulon*, Sect. *Cephalophilon*, and *P. kawagoeana*. In this study, *Polygonum aviculare* (NC058892) served as an outgroup, while chloroplast genome sequences from 25 published *Persicaria* species, obtained from the NCBI database, were utilized to construct a phylogenetic tree with *P. criopolitana*. The chloroplast genome of *P. criopolitana* and other Polygonaceae species was analyzed using the multi-sequence comparison tool MAFFT^56^. Maximum likelihood (ML) and Bayesian inference (BI) tree models were calculated with the ModelFinder tool in PhyloSuite v.1.2.2^57^. For constructing the ML tree, RAxML v.7.2.8^58^ was employed, while MrBayes v.3.1.2^59^ was used for the BI tree.

## Data Availability

The complete chloroplast genomes and annotations are available at the NCBI database (*Persicaria criopolitana*: PQ858440).
